# Effect of Long-Term and Short-Term Imbalanced Zn Manipulation on Gut Microbiota and Screening for Microbial Markers Sensitive to Zinc Status

**DOI:** 10.1128/Spectrum.00483-21

**Published:** 2021-11-03

**Authors:** Lingjun Chen, Zhonghang Wang, Peng Wang, Xiaonan Yu, Haoxuan Ding, Zinan Wang, Jie Feng

**Affiliations:** a Key Laboratory of Molecular Animal Nutrition, Ministry of Education, College of Animal Sciences, Zhejiang University, Hangzhou, China; b Elpida Institute of Life Sciences, Hangzhou, Zhejiang, China; Huazhong University of Science and Technology

**Keywords:** dietary zinc, intestinal microbiome, short-chain fatty acids, zinc biomarker

## Abstract

Zinc (Zn) imbalance is a common single-nutrient disorder worldwide, but little is known about the short-term and long-term effects of imbalanced dietary zinc in the intestinal microbiome. Here, 3-week-old C57BL/6 mice were fed diets supplemented with Zn at the doses of 0 (low Zn), 30 (control Zn), 150 (high Zn), and 600 mg/kg of body weight (excess Zn) for 4 weeks (short term) and 8 weeks (long term). The gut bacterial composition at the phyla, genus, and species levels were changed as the result of the imbalanced Zn diet (e.g., Lactobacillus reuteri and Akkermansia muciniphila). Moreover, pathways including carbohydrate, glycan, and nucleotide metabolism were decreased by a short-term low-Zn diet. Valeriate production was suppressed by a long-term low-Zn diet. Pathways such as drug resistance and infectious diseases were upregulated in high- and excess-Zn diets over 4-week and 8-week intervals. Long-term zinc fortification doses, especially at the high-Zn level, suppressed the abundance of short-chain fatty acids (SCFAs)-producing genera as well as the concentrations of metabolites. Finally, *Melainabacteria* (phylum) and *Desulfovibrio* sp. strain ABHU2SB (species) were identified to be potential markers for Zn status with high accuracy (area under the curve [AUC], >0.8). Collectively, this study identified significant changes in gut microbial composition and its metabolite concentration in altered Zn-fed mice and the relevant microbial markers for Zn status.

**IMPORTANCE** Zn insufficiency is an essential health problem in developing countries. To prevent the occurrence of zinc deficit, zinc fortification and supplementation are widely used. However, in developed countries, the amounts of Zn consumed often exceed the tolerable upper intake limit. Our results demonstrated that dietary Zn is an essential mediator of microbial community structure and that both Zn deficiency and Zn overdose can generate a dysbiosis in the gut microbiota. Moreover, specific microbial biomarkers of Zn status were identified and correlated with serum Zn level. Our study found that a short-term low-Zn diet (0 mg/kg) and a long-term high-zinc diet (150 mg/kg) had obvious negative effects in a mouse model. Thus, these results indicate that the provision and duration of supplemental Zn should be approached with caution.

## INTRODUCTION

Zinc is an essential nutrient for nearly all organisms (1). The World Health Organization (WHO) reported that at least 30% of the world population is affected by inadequate zinc, especially for children in developing countries (2). Zn deficiency may induce growth retardation, hypogonadism (3), diarrhea (4), or acrodermatitis enteropathica (AE) (5). Hence, additional zinc is often given to children in foods, drinks, and nutritional supplements to ensure that they meet their physiological requirements for growth (6). Nevertheless, the amounts of Zn consumed often exceed well-established nutritional requirements (7–9). Recent studies reported that Zn overdose exposure can have deleterious consequences, such as decreasing immune function, altering intestinal absorptive and secretory capacity, or increasing visceral adiposity (10–12).

The human gut microbiome has emerged as a major component in host health status over the past decade (13). It serves several important functions, such as enhancement of immunity and prevention of allergens and generation of SCFAs (14). Alterations in dietary zinc intake may play a major role in community dynamics and interspecies competition in the intestine. Zn deficiency may increase susceptibility to bacterial infection, such as Brucella abortus, Salmonella enterica, and Campylobacter jejuni (15–17). Little is known about how Zn overdose affects the intestinal flora. Zackular et al. have shown that in the mouse model of C. difficile infection, excess dietary Zn alters the gut microbiota and decreases resistance to C. difficile infection (CDI) (18). So far, little is known about the short-term or long-term efficacy of imbalanced dietary zinc in the intestinal microbiome in young animals and children.

Currently, the lack of a reliable, responsive, and specific zinc biomarker has made the quantification and characterization of Zn imbalance difficult (19). Lowe and coworkers suggested that serum, plasma, hair, and urinary zinc were the most reliable predictors out of 32 potential biomarkers in humans (20). However, accurate assessment of zinc status, especially in marginal imbalance, is difficult, as studies have shown contradictory and inconsistent results. To solve this problem, the WHO and the International Zinc Nutrition Consultative Group (IZiNCG) have put forward a major initiative to promote the development of reliable Zn biomarkers (21). Host lifestyle and behaviors can be reflected by human gut microbiome variation (22). Moreover, dietary metal intake is one of the most critical factors affecting the composition of the microbiome (23). Previous findings based on the human gut have enabled the tentative prediction of urinary arsenic, coronavirus disease 2019 (COVID-19), and adiposity (24–26).

In order to clarify the relationship between dietary zinc and intestinal microflora, we fed different zinc levels to mice from weaning to puberty or maturity to explore the effects of zinc imbalance on intestinal microflora and their metabolites (SCFAs) and screened the microbial markers sensitive to zinc status.

## RESULTS

### The effect of dietary zinc on growth performance and colonic morphology.

**FIG 1:**
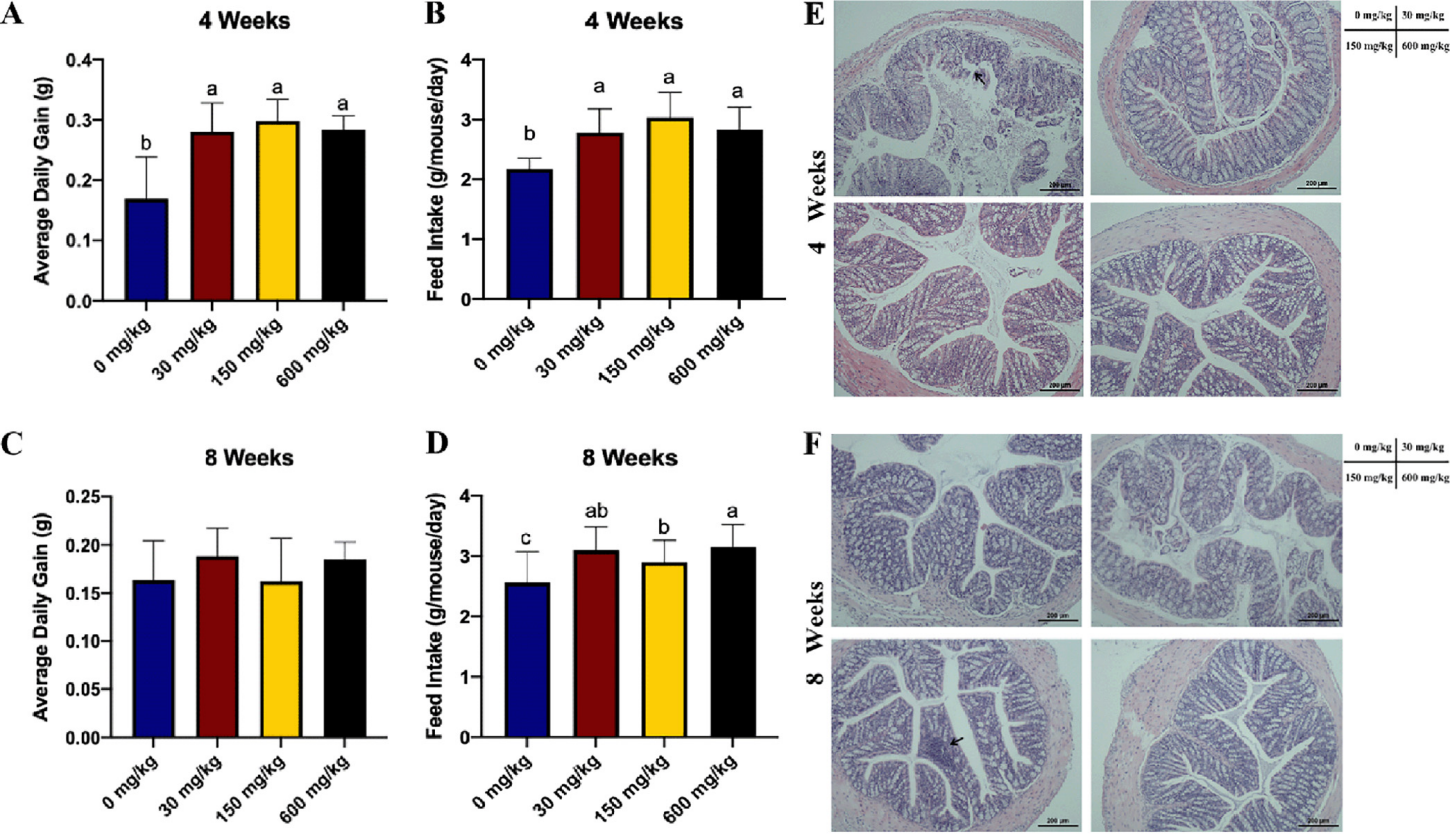
Effect of imbalanced dietary Zn on growth performance and colonic morphology in mice at different stages. (A) Daily weight gain and (B) feed intake in the 4-week Zn intervention (*n* = 8 per group). (C) Daily weight gain and (D) feed intake in the 8-week Zn intervention (*n* = 8 per group). (E) Representative H&E staining images of colonic tissue sections in the 4-week Zn intervention; the arrow denotes a slight shedding of colon villus. (F) Representative H&E staining images of colonic tissue sections in the 8-week Zn intervention; the arrow denotes a partial colon inflammatory cell infiltration. Groups labeled without a common letter were significantly different (*P* < 0.05).

To determine the effect of imbalanced dietary Zn and the intervention duration on weaning mice, growth parameters were monitored during the experiment. During the 4-week Zn intervention (short-term zinc intervention), the average daily gain and feed intake were significantly decreased with a low-Zn diet (0 mg of Zn/kg of body weight) (Fig. 1A and B). During the 8-week Zn intervention (long-term zinc intervention), the results for the average daily gain were similar among the four groups (Fig. 1C). In comparison with the control-Zn group (30 mg/kg), a marked decrease in the feed intake was observed in both the low-Zn and high-Zn (150 mg/kg) groups (Fig. 1D).

Distal colon samples were stained with hematoxylin and eosin (H&E) to examine morphological changes due to Zn levels and life stage. A slight shedding of colon villus was observed in the 4-week low-Zn group (Fig. 1E), and a partial colon inflammatory cell infiltration was observed in the 8-week high-Zn group (Fig. 1F).

### The effect of dietary zinc on gut microbial diversity.

**FIG 2:**
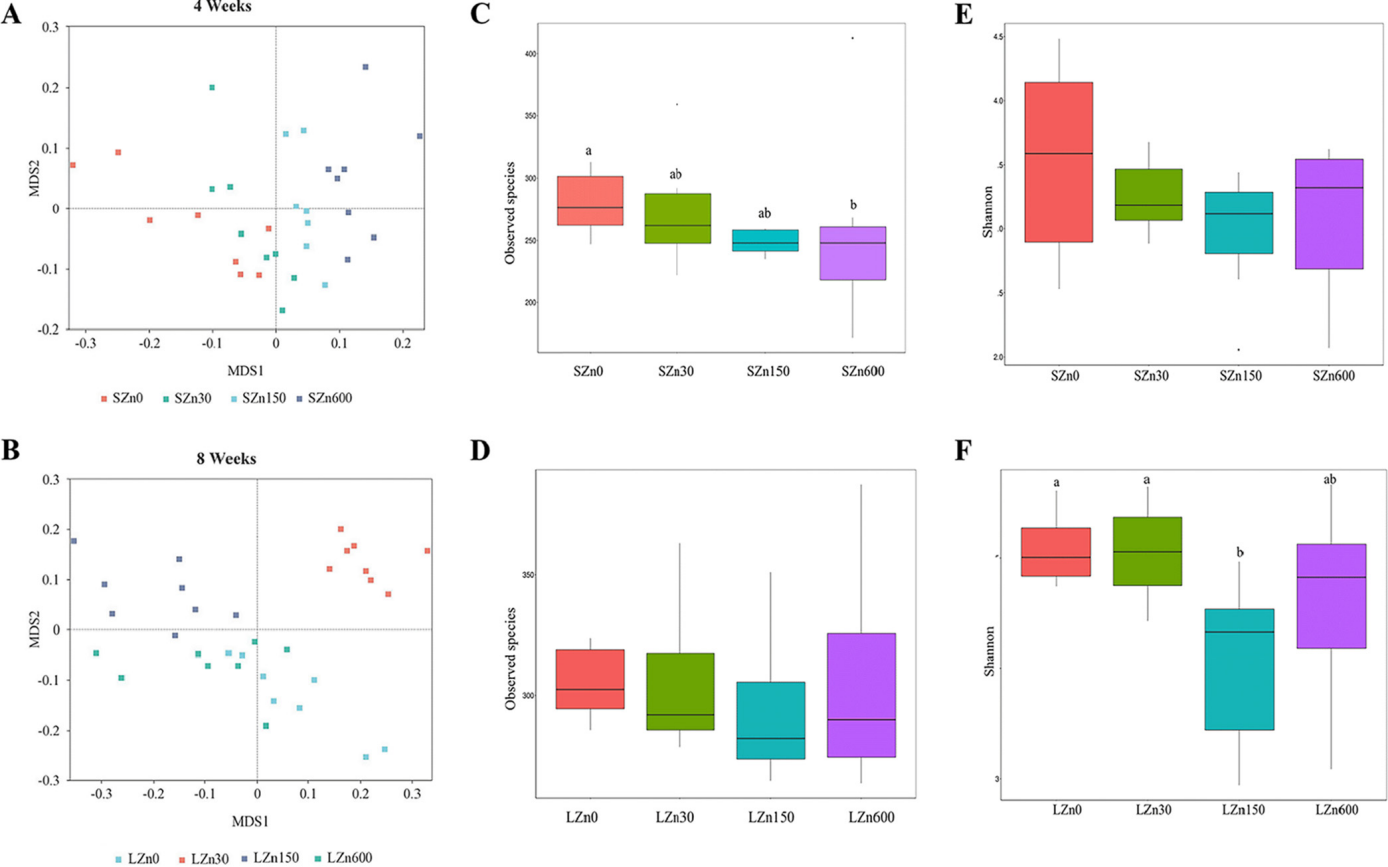
Imbalanced dietary Zn dramatically alters the gut microbiota. Nonmetric multidimensional scaling (NMDS), based on the Bray-Curtis distances at the OTU level. (A) Ordination plot showing cecal microbiota β-diversity of mice from the short-term 0-mg/kg Zn intervention (SZn0), short-term 30-mg/kg Zn intervention (SZn30), short-term 150-mg/kg Zn intervention (SZn150), and short-term 600-mg/kg Zn intervention (SZn600). (B) NMDS showed distinct clusters of gut bacteria in mice from the long-term 0-mg/kg Zn intervention (LZn0), long-term 30-mg/kg Zn intervention (LZn30), long-term 150-mg/kg Zn intervention (LZn150), and long-term 600-mg/kg Zn intervention (LZn600). (C to F) Measures of α-diversity using the total number of observed species in (C) 4-week and (D) 8-week time courses of dietary Zn manipulation and Shannon index in the (E) 4-week Zn intervention and (F) 8-week Zn intervention.

Mice fed with altered-Zn diets for 4 weeks or 8 weeks both showed a marked shift in microbial community structure compared to that in control-Zn-fed mice (Fig. 2A and B and Table S1 in the supplemental material). In the short-term zinc intervention, there was a significant decrease in observed species for the excess-Zn-fed mice (600 mg/kg) compared with that of the low-Zn-fed mice, whereas no clear differences were observed in the Shannon index (Fig. 2C and E). In the long-term zinc intervention, the observed species were similar among the four groups. A higher Shannon index was generally observed in the low-Zn group and control-Zn group, whereas the high-Zn group showed the lowest Shannon index (Fig. 2D and F).

### The effect of dietary zinc on gut microbial composition at the phylum level.

**FIG 3:**
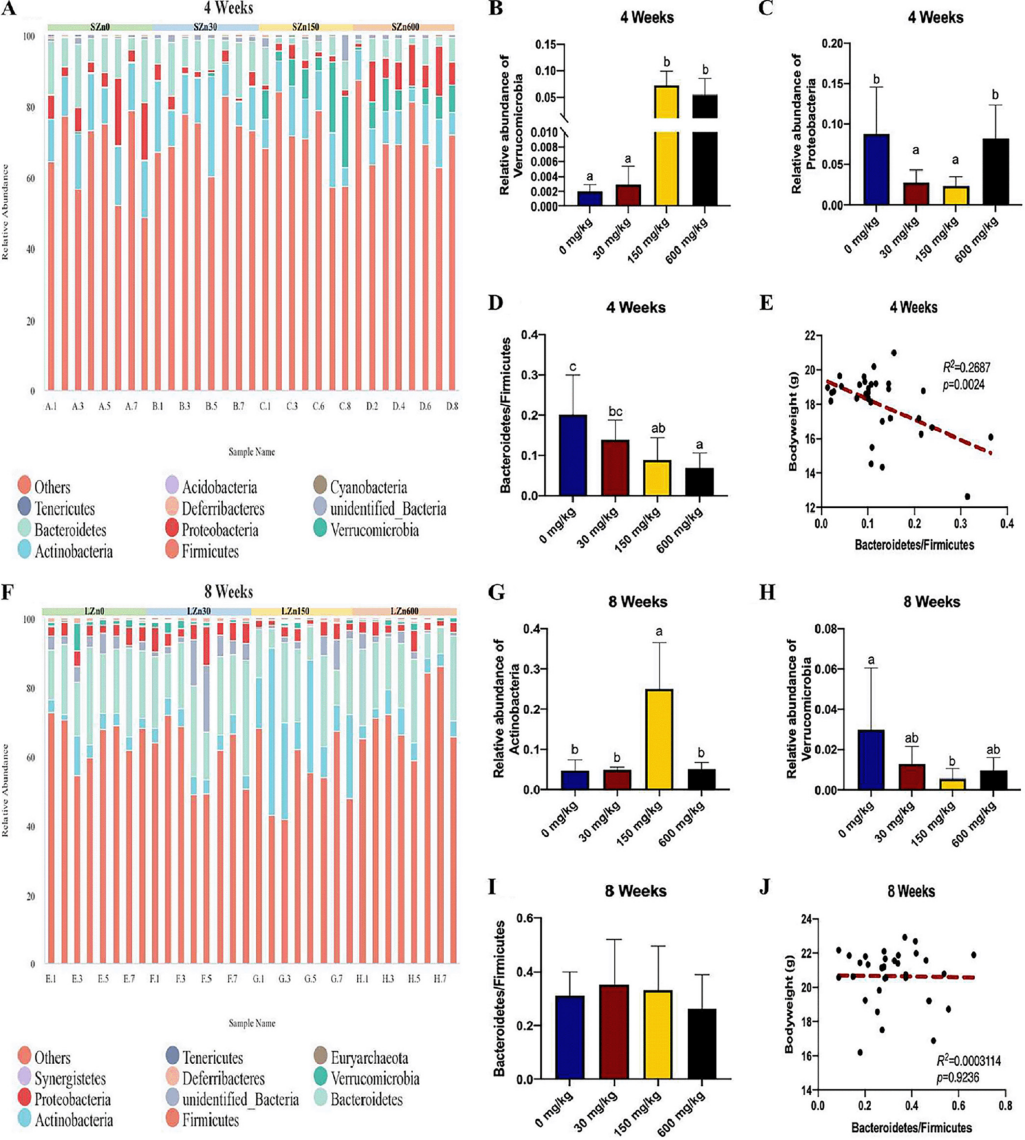
Imbalance of dietary zinc disturbs the microbial community structure in the mouse cecum at the phylum level. (A) The top 10 microbial populations at the phylum level in the cecum of mice from the short-term 0-mg/kg Zn intervention (SZn0), short-term 30-mg/kg Zn intervention (SZn30), short-term 150-mg/kg Zn intervention (SZn150), and short-term 600-mg/kg Zn intervention (SZn600). (B) Mice on the 150-mg/kg- and 600-mg/kg Zn diet in 4 weeks show increased *Verrucomicrobia* levels. (C) Mice on Zn-deficient and Zn-excessive diets had significantly higher levels of *Proteobacteria*. (D) During the short-term intervention, the ratio of *Firmicutes*/*Bacteroidetes* was positively related with Zn dosage. (E) Linear correlation between bodyweight and *Firmicutes*/*Bacteroidetes* ratio after 4 weeks. (F) Relative abundance (top 10) of bacterial phyla in the cecum of mice from the long-term 0-mg/kg Zn intervention (LZn0), long-term 30-mg/kg Zn intervention (LZn30), long-term 150-mg/kg Zn intervention (LZn150), and long-term 600-mg/kg Zn intervention (LZn600). (G) Increased *Actinobacteria* level in long-term 150-mg/kg Zn-fed mice. (H) Long-term Zn-starvation mice had an increased abundance of *Verrucomicrobia*. (I) *Firmicutes*/*Bacteroidetes* ratio in altered-Zn diets. (J) Correlation analysis between body weight and *Firmicutes*/*Bacteroidetes* ratio after 8 weeks. Groups labeled without a common letter were significantly different (*P* < 0.05).

Changes in the relative abundance of phylum populations explained the differences in community structure between the short-term and long-term Zn interventions (low-Zn diet, control-Zn diet, high-Zn diet, and excess-Zn diet, respectively). *Firmicutes* (65.7, 72.4, 69.7, and 71.9%), *Actinobacteria* (13.9, 13.9, 10.7, and 9%), *Verrucomicrobia* (0.2, 0.3, 10.2, and 5.4%), *Proteobacteria* (7.8, 2.8, 2.1, and 8.2%), and *Bacteroidetes* (11.3 9.3, 5.2, and 4.7%) were the most predominant phyla in the cecum of mice that were fed with altered zinc diets for 4 weeks, accounting for more than 97.9% of the total sequences (Fig. 3A). Minor members of the community were affiliated with the *Deferribacteres* (0.26, 0.1, 0.14, and 0.03%), the *Tenericutes* (0.049, 0.005, 0.003, and 0.002%), the *Cyanobacteria* (0.002, 0.007, 0, and 0.002%), and the *Acidobacteria* (0.003, 0.0008, 0.004, and 0.002%). The high-Zn diet and excess-Zn diet substantially enhanced the abundance of the *Verrucomicrobia* among the four groups (Fig. 3B), and the proportion of the *Proteobacteria* was significantly higher in the low-Zn diet and excess-Zn diet than in the control and high-Zn diets (Fig. 3C). The decrease in the ratio of *Bacteroidetes*:*Firmicutes* was accompanied by the increase of Zn dosage (Fig. 3D). Additionally, the ratio of *Bacteroidetes*:*Firmicutes* was inversely correlated with body weight (Fig. 3E).

In 8-week samples, *Firmicutes* (65.5, 60.2, 55, and 71.2%), *Actinobacteria* (4.5, 4.7, 21.6, and 4.9%), *Bacteroidetes* (20.1, 20.9, 16.6, and 17.5%), *Proteobacteria* (3.6, 4.5, 2.8, and 3%), and *Verrucomicrobia* (2.1, 1.7, 0.56, and 0.96%) occupied more than 92.4% of the total sequences (Fig. 3F). Minor members of the community were *Deferribacteres* (0.7, 0.58, 0.32, and 0.22%), *Synergistetes* (0.012, 0.08, 0.07, and 0.08%), *Euryarchaeota* (0.03, 0.06, 0.09, and 0.0745%), and *Tenericutes* (0.005, 0.016, 0.05, and 0.004%). The highest abundance of *Actinobacteria* was observed in mice fed high-Zn diets (Fig. 3G). In contrast to the result of short-term Zn supplementation, the proportion of *Verrucomicrobia* was increased in the low-Zn group and was decreased in the high-Zn group (Fig. 3H). The ratio of *Bacteroidetes*:*Firmicutes* showed insignificant differences among the four groups and was not significantly correlated with the body weight (Fig. 3I and J).

### The effect of dietary zinc on gut microbial composition at the genus level.

Analysis of the relative abundance of the dominant genera (top 30) revealed the clear and distinct clustering of cecal microbiota in altered Zn diets in short-term and long-term Zn intervention (Fig. S1). During the short-term Zn intervention, the cecal contents of mice fed the low-Zn diet were characterized by an increase in the relative abundance of *Desulfovibrio*, *Enterorhabdus*, *Flavonifractor*, *Parasutterella*, *Mucispirillum*, “*Candidatus* Saccharimonas,” *Alistipes*, *Odoribacter*, and *Anaerotruncus*, and a decrease in the relative abundance of *Sphingomonas*, compared with the other dietary treatments (Fig. S1A). The relative abundance of unidentified *Lachnospiraceae*, *Lachnoclostridium*, and *Bifidobacterium* in the control-Zn group was higher than that in the high- and excess-Zn groups. The high-Zn diet was associated with enrichment of *Akkermansia*, *Faecalibaculum*, *Helicobacter*, and *Ileibacterium* compared to other diets. Mice fed the 600 mg/kg Zn diet showed a higher abundance of *Dubosiella*, *Caulobacter*, and *Bradyrhizobium* and a lower proportion of *Romboutsia*, *Bacteroides*, *Lactobacillus*, and *Bifidobacterium*.

Long-term Zn alteration reversed the distribution of some bacterial genera compared with short-term Zn intervention (Fig. S1B). Under low-Zn conditions, an increase in the relative abundance of *Akkermansia*, *Blautia*, *Alloprevotella*, and *Ruminiclostridium* and a decrease in *Thermovirga* were found in cecum of mice. The relative abundance of *Parasutterella*, *Helicobacter*, *Odoribacter*, and *Ileibacterium* in the control-Zn group was higher and the proportion of *Dubosiella*, *Faecalibaculum*, and *Bifidobacterium* was lower than in the other groups. For the high-Zn-diet group, most genera were decreased compared with the other dietary treatments, except for *Bifidobacterium* and *Anaeroplasma*, which were increased. Mice fed the excess-Zn diet showed increases in the level of *Bacteroides* and *Intestinimonas* and decreases in *Lactobacillus*.

### The effect of dietary zinc on gut microbial composition at the species level.

**FIG 4:**
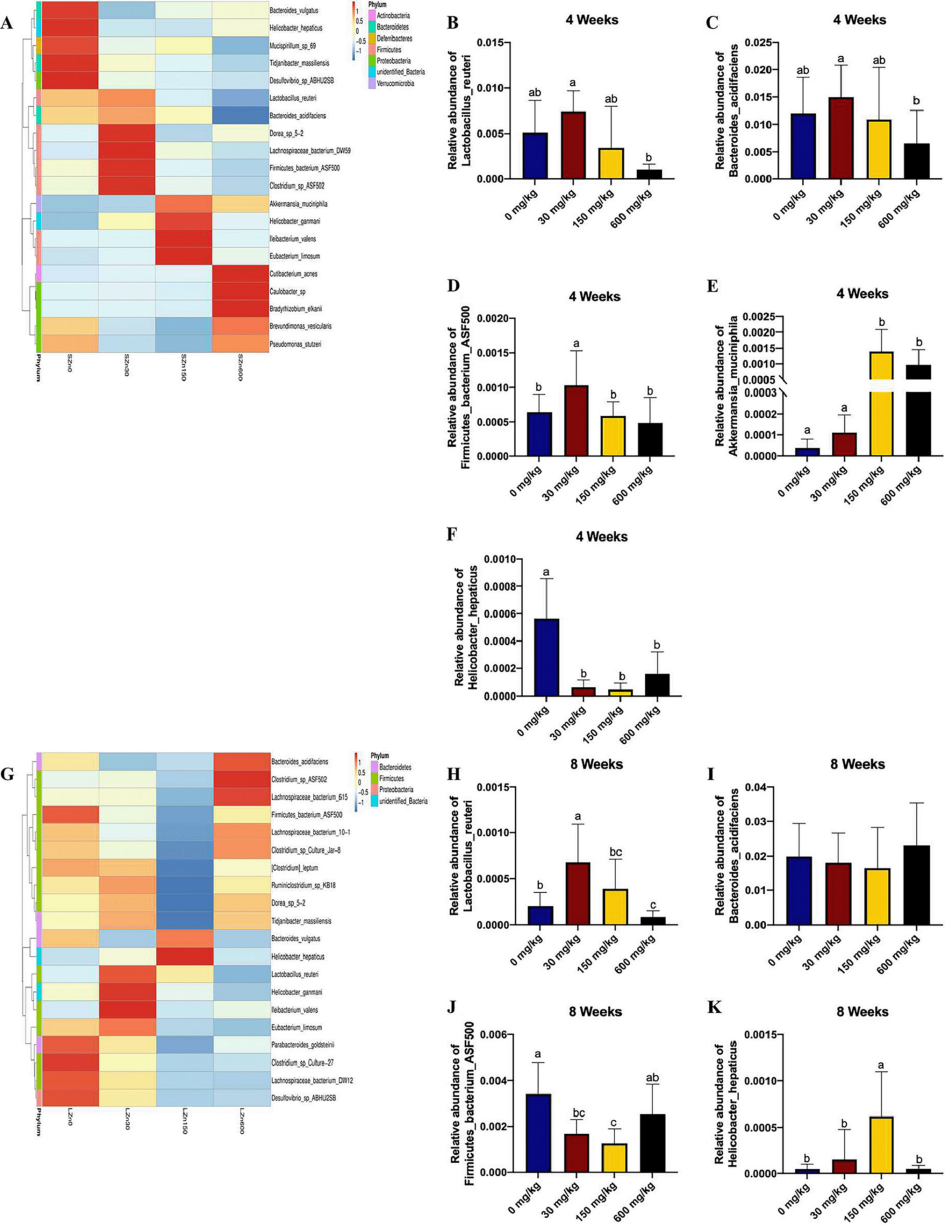
Imbalance of dietary zinc disturbs the microbial community structure in the mouse cecum at the species level. (A) The top 20 microbial populations at the species level in the cecum of mice from the short-term 0-mg/kg Zn intervention (SZn0), short-term 30-mg/kg Zn intervention (SZn30), short-term 150-mg/kg Zn intervention (SZn150), and short-term 600-mg/kg Zn intervention (SZn600). (B) Lactobacillus reuteri was significantly augmented in the short-term excessive-Zn diet. (C) Relative abundance of *Firmicutes bacterium* ASF500 in the 4-week time course of dietary Zn manipulation. (D) During the short-term intervention, the proportion of Bacteroides acidifaciens was significantly increased in control-Zn diet and decreased in low-Zn and excess-Zn diets. (E) The abundance of Akkermansia muciniphila was increased in 150-mg/kg and 600-mg/kg Zn-fed mice. (F) The Zn-starvation mice showed the enrichment of Helicobacter hepaticus. (G) Relative abundance (top 20) of bacterial species in the cecum of mice from the long-term 0-mg/kg Zn intervention (LZn0), long-term 30-mg/kg Zn intervention (LZn30), long-term 150-mg/kg Zn intervention (LZn150), and long-term 600-mg/kg Zn intervention (LZn600). (H) Increased Lactobacillus reuteri level in long-term 30-mg/kg Zn-fed mice. (I) Relative abundance of Bacteroides acidifaciens in the 8-week time course of dietary Zn manipulation. (J) The low-Zn diet and excess-Zn diet were associated with the enrichment of *Firmicutes bacterium* ASF500. (K) The abundance of Helicobacter hepaticus was increased in 150-mg/kg Zn-fed mice. Groups labeled without a common letter were significantly different (*P* < 0.05).

We further assessed differences in the bacterial community at the species level (potential probiotics and pathobionts among top the 20 species) (Fig. 4A and G). In the 4-week samples, the abundance of Lactobacillus reuteri and *Firmicutes bacterium* ASF500 was significantly increased in the control-Zn diet compared with other diets, and the relative abundance of Bacteroides acidifaciens was decreased in excess-Zn-diet fed mice compared to control-Zn-fed mice (Fig. 4B to D). High- and excess-Zn supplementation substantially enhanced the abundance of Akkermansia muciniphila in the cecum of mice (Fig. 4E). The low-Zn diet showed the enrichment of Helicobacter hepaticus compared with the other dietary treatments (Fig. 4F).

In the 8-week samples, the highest abundance of Lactobacillus reuteri was observed in the control-Zn diet, whereas the abundance of Bacteroides acidifaciens was similar among the four groups (Fig. 4H and I). The low-Zn diet and excess-Zn diet were both associated with the enrichment of *Firmicutes bacterium* ASF500 (Fig. 4J). The abundance of Helicobacter hepaticus was higher in the high-Zn group than in the other groups (Fig. 4K).

### Microbiota function prediction.

**FIG 5:**
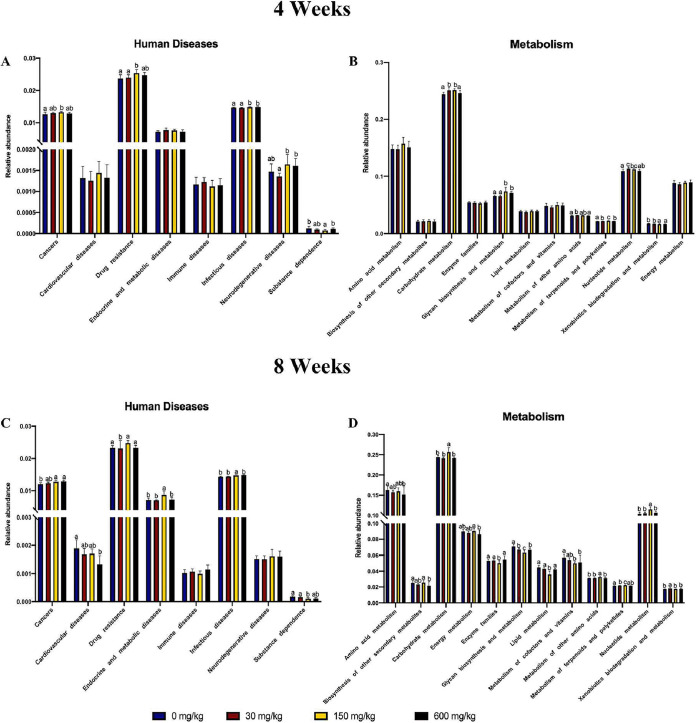
Imbalanced dietary zinc shapes the microbial functional capacity in the mouse cecum. Tax4Fun-generated graphs showing predicted functional capabilities of microbial communities based on 16S rRNA data sets. (A and B) In the 4-week Zn intervention, the relative abundance of significantly affected KEGG pathways related to (A) human diseases and (B) metabolism. (C and D) In the 8-week Zn intervention, the relative abundance of significantly affected KEGG pathways related to (C) human diseases and (D) metabolism. Groups labeled without a common letter were significantly different (*P* < 0.05).

Analysis of functional capacity based on the KEGG pathways “human diseases” and “metabolism” showed significant differences in altered dietary Zn groups (Fig. 5). During the short-term Zn intervention, genes related to human diseases showed a significantly higher abundance in the high-Zn group and a remarkably lower abundance in the control-Zn group for a high number of genes involved, i.e., in cancers, drug resistance, infection disease, and neurodegenerative diseases (Fig. 5A). Of note, a pathway involved in substance dependence was increased in the low-Zn group and the excess-Zn group, while it was decreased in the high-Zn group. Among all the metabolism pathways analyzed, the level of carbohydrate metabolism, glycan biosynthesis and metabolism, metabolism of other amino acids, metabolism of terpenoids and polyketides, and nucleotide metabolism were suppressed in the low-Zn diet and promoted in the high-Zn diet (Fig. 5B). A higher proportion of genes related to xenobiotic biodegradation and metabolism was observed in both the low-Zn group and the control-Zn group than in the other two higher-Zn-fed groups.

During the long-term Zn intervention, the high-Zn-fed mice were enriched in human disease pathways involved in cancers, drug resistance, endocrine and metabolic diseases, and infectious diseases and were depleted in the gene related to substance dependence (Fig. 5C). Genes related to cardiovascular diseases showed a significantly higher abundance in the low-Zn group and a lower abundance in the excess-Zn group. In terms of metabolism pathways, the relative abundances of amino acid metabolism and biosynthesis of other secondary metabolites were dramatically increased in the low-Zn group and decreased in the excess-Zn group (Fig. 5D). Mice fed the high-Zn diet had increased genes related to carbohydrate metabolism, energy metabolism, and nucleotide metabolism and decreased genes involved in enzyme families, lipid metabolism, metabolism of cofactors and vitamins, and metabolism of terpenoids and polyketides. The higher proportions of the gene for xenobiotic biodegradation and metabolism were observed with the control-Zn diet.

### The effect of dietary zinc on metabolites.

**FIG 6:**
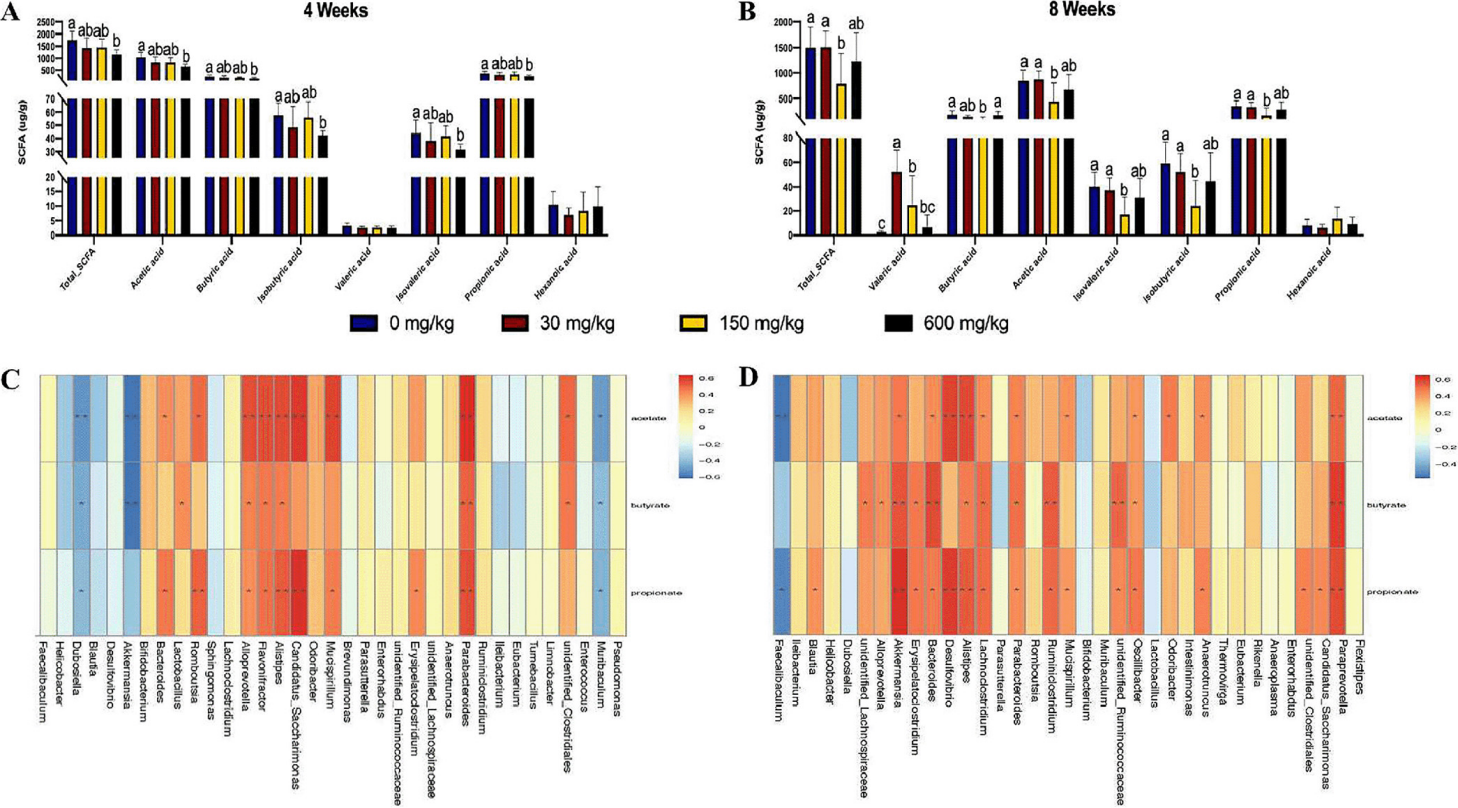
Imbalanced dietary zinc alters the metabolites in the mouse cecum. (A and B) Concentration of metabolites in the cecal contents in altered-Zn diets in (A) 4 weeks and (B) 8 weeks. (C and D) Heat maps describing a set of Spearman correlations, independent of treatment group, between altered genus and cecal acetate, propionate, and butyrate status in (C) the 4-week Zn intervention and (D) the 8-week intervention. The colors range from a perfect negative correlation (–0.6, blue) to a perfect positive correlation (0.6, red) (* *P < *0.05; ** *P < *0.01; ANOVA).

Analysis of the total and individual metabolites revealed significant differences between dietary groups. During the short-term Zn intervention, the lowest levels of total metabolites were determined in the excess-Zn group, followed by the control-Zn group, high-Zn group, and low-Zn group in that order. Interestingly, a highly similar pattern was also observed for total SCFAs, acetic acid, butyric acid, isobutyric acid, isovaleric acid, and propionic acid (Fig. 6A).

During the long-term Zn intervention, high-Zn diet resulted in a marked loss of cecal levels of total metabolites compared with the low-Zn diet and control-Zn diet, as well as total SCFAs, butyric acid, acetic acid, isovaleric acid, isobutyric acid, and propionic acid. The highest concentration of valeric acid was observed with the control-Zn diet, followed by the high-Zn and excess-Zn diets, while the low-Zn diet showed the lowest proportion (Fig. 6B).

On the basis of the data for Zn-treated mice, we hypothesized that SCFAs were markedly influenced by microbiota alterations. *Enterorhabdus*, *Alistipes*, *Odoribacter*, *Anaerotruncus*, *Alloprevotella*, *Bacteroides*, and *Parabacteroides* were changed both in 4-week and 8-week courses of Zn manipulation, and all of them belong to short-chain fatty acid-producing genera (27–29). Therefore, correlation analyses between the dominant genera and SCFAs, primarily acetate, propionate, and butyrate, were conducted by Spearman correlation analysis (Fig. 6C and D). Notably, cecal SCFAs production showed remarkable correlation with the relative abundances of *Bacteroides*, *Alistipes*, and *Parabacteroides*, which indicates that these three genera mainly contributed to SCFAs production in this study.

### Prediction of imbalanced Zn based on bacterial markers in gut.

**FIG 7:**
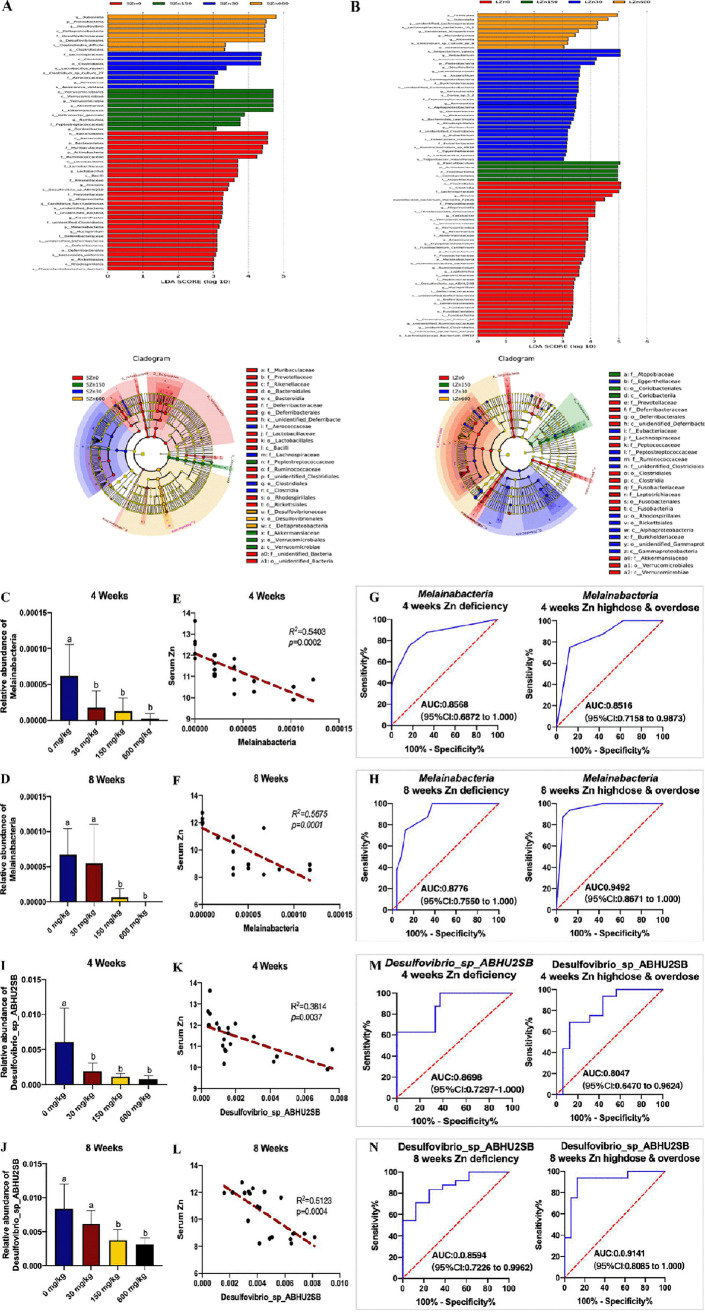
Prediction of imbalanced Zn based on bacterial markers in the gut. (A and B) Linear discriminant analysis (LDA) effect size (LEfSe) plot of taxonomic biomarkers identified in the gut microbiome of mice in (A) 4-week and (B) 8-week time courses of dietary Zn manipulation. The LEfSe algorithm, emphasizing both statistical and biological relevance, was used for biomarker discovery. The threshold for the linear discriminant analysis (LDA) score was 3. (C and D) The relative abundance of *Melainabacteria* in the altered-Zn groups in (C) the 4-week and (D) the 8-week intervention. (E and F) Correlation analysis between the proportion of *Melainabacteria* and serum Zn levels in (E) 4 weeks and (F) 8 weeks. (G and H) Prediction (Zn deficiency/Zn high dose and overdose) of *Melainabacteria* in the microbiome of altered-Zn-fed mice in (G) 4 weeks and (H) 8 weeks. (I and J) The relative abundance of *Desulfovibrio* sp. ABHU2SB in the altered-Zn groups in (I) the 4-week and (J) the 8-week intervention. (K and L) Correlation analysis between the proportion of *Desulfovibrio* sp. ABHU2SB and serum Zn levels in (K) 4 weeks and (L) 8 weeks. (M and N) Prediction (Zn deficiency/Zn high dose and overdose) of *Desulfovibrio* sp. ABHU2SB in the microbiome of altered-Zn-fed mice in (M) 4 weeks and (N) 8 weeks. The area under the receiver operating characteristic curve (ROC [blue curve]), with 95% confidence interval (CI), is shown in the center.

To document the impact of imbalanced Zn exposure on the composition of gut microbiota, we performed linear discriminant analysis effect size (LEfSe) analyses using the relative abundance data to identify the bacteria differentially represented among different dietary Zn manipulations. There were 55 differential bacterial taxa in the short-term Zn intervention samples and 79 differential bacterial taxa in the long-term treatment samples (Fig. 7A and B). At the phylum level, LEfSe analysis identified *Melainabacteria* as a strongly discriminative taxon for both short-term and long-term dietary Zn intervention (Fig. 7A to D). Next, correlation analysis between *Melainabacteria* and serum Zn were conducted by Pearson correlation analysis (Fig. 7E and F). As expected, the serum Zn levels were negatively correlated with the relative abundance of *Melainabacteria* at both 4 and 8 weeks (*P* = 0.0002 and *P* = 0.0001, respectively). Receiver operating characteristic (ROC) analysis implied that the level of *Melainabacteria* could potentially be applied to differentiate Zn levels with high accuracy (AUC, >0.85, Fig. 7G and H). In terms of genus level, *Lactobacillus* was more abundant in the low-Zn group over the 4- and 8-week intervals. *Lactobacillus* attained good diagnostic accuracy to distinguish Zn deficiency and Zn overdose over the 4-week interval (AUC, >0.84). However, in 8 weeks, this genus was not significantly correlated with the serum Zn, and the AUC was only 0.69 (Fig. S2A-F). Our LEfSe analysis also identified a species-level bacterium (*Desulfovibrio* sp. strain ABHU2SB) that was specific for different dietary Zn levels (Fig. 7I and J). According to the Pearson correlation analysis and ROC curve, the serum Zn levels are highly correlated with the relative abundance of *Desulfovibrio* sp. ABHU2SB over the 4- and 8-week intervals (*P* = 0.0037 and *P* = 0.0004, respectively), with a ROC plot AUC value of 0.8 to 0.91 (Fig. 7K to N).

## DISCUSSION

The analysis of the microbial communities has revealed the dramatic alteration of the gut microbiota in conditions of imbalanced dietary Zn. The low-Zn diet did not decrease the intestinal species richness, which is in line with the previous finding in mice (18). Intriguingly, Zn starvation showed statistically significant decreased microbial diversity in broilers and piglets (30, 31). A possible explanation for this difference is the animal model used. In livestock production, Zn-supplemented diets are regularly utilized to administer drugs, provide nutrients and prevent deficiencies, and increase the size and output of animals (32). We speculated that livestock was more sensitive to Zn-limited condition than mice. Therefore, Zn starvation might be insufficient for any significant decrease in the microbial diversity in mice. The shift in community composition after Zn addition was accompanied by a reduction in alpha diversities over the 4- and 8-week intervals, supporting the previous finding in mice (18). Furthermore, pharmacological intakes of ZnO (2500 mg Zn/kg) have been also been shown to affect the regulation of the microbial communities by reducing the microbial richness in the jejunum and feces (33) or the ileal digesta of weaned piglets (34).

The altered-Zn-diet intervention resulted in notable changes in the bacterial composition at the phylum, genus, and species levels. An increased prevalence of *Proteobacteria* is a potential diagnostic signature for dysbiosis or risk of disease (35). During a feeding period of 4 weeks, mice on low-Zn and excess-Zn diets showed a remarkable increase in the phylum *Proteobacteria*, as well as the genus *Desulfovibrio*, a potential biomarker in inflammatory bowel diseases (36). The zinc repressor ZUR, which regulates high-affinity zinc transporters znuABC, has been found in *Desulfovibrio* species (37). Therefore, a lack of sufficient dietary Zn in the cecum may modulate the level of *Desulfovibrio* by enabling colonization and outgrowth of Zn-competing bacteria. Furthermore, a heavy metal resistance gene (*tetQ*) was found to be affiliated with *Proteobacteria* (38), it is likely that this phylum possesses certain mechanisms to counteract the high dietary zinc. Therefore, the phylum *Proteobacteria* was determined to possess a good adaptive capacity under imbalanced zinc conditions. However, dietary Zn alteration did not significantly affect the level of *Proteobacteria* in the 8-week intervention. This could be rationalized by the fact that the intestinal flora of mice would stabilize gradually with age (39), which makes it easier to prevent the colonization of potential pathogens. Akkermansia muciniphila, a member of the *Verrucomicrobia*, is an important gut symbiont for the maintenance of metabolic homeostasis and is associated with low inflammation and normal lipid and carbohydrate metabolism (40). In our study, the proportion of *Verrucomicrobia* was increased in mice that were fed high- and excess-Zn diets and was decreased in Zn-starvation mice over the 4-week interval. Statistically significant differences also were noted at the genus (*Akkermansia*) and species (Akkermansia muciniphila) levels. This is in agreement with the previous studies showing that zinc deficiency is correlated with low relative abundances of *Verrucomicrobia* (41, 42). Furthermore, the percentages of *Verrucomicrobia* was significantly higher in the 3,000 mg/kg ZnO group of weaning piglets (43). In the 8-week Zn intervention, *Verrucomicrobia* was found at higher abundance in the low-Zn group and lower abundance in the high-Zn group. This phenomenon revealed that a high dose of Zn has a profound effect on *Verrucomicrobia*; whether it is positive or negative depends on the exposure duration. We hypothesized that long-term Zn accumulation could be highly toxic to *Verrucomicrobia*, which may occur through intermetal competition, mis-metallation of metalloprotein, or redox activity (44). More *in vitro* and *in vivo* studies are needed to determine how zinc may affect the growth of *Verrucomicrobia* (*A. muciniphila*), and how they may interact in the process. Lower *Bacteroidetes*:*Firmicutes* ratios have been associated with several disorders, such as human obesity (45), inflammatory bowel disease (IBD; 46), and type 2 diabetes (47). In our study, during the short-term interventions, the ratio of *Bacteroidetes*:*Firmicutes* was negatively related to the Zn dosage. In agreement with previous literature, a significantly greater abundance of *Bacteroidetes* and a significantly lower abundance of *Firmicutes* was observed in the Zn-deficient group (30). In addition, the phylum *Firmicutes* thrived under low-zinc conditions in pregnant mice (48). However, long-term Zn intervention did not significantly influence the ratio of *Bacteroidetes*:*Firmicutes*. It was assumed that the relative proportional representation of the *Firmicutes* and *Bacteroidetes* may play a role in the development of body weight gain (49). Consistent with the ratio of *Bacteroidetes*/*Firmicutes*, there is no significant alteration of body weight gain among different levels of Zn diets in 8 weeks. We reasoned that this substantial difference (*Bacteroidetes*:*Firmicutes*) in long-term and short-term Zn manipulation may be linked not only to intestinal Zn accumulation duration but also to the operation of age, as Mariat and coworkers demonstrated that the ratio of *Firmicutes* to *Bacteroidetes* evolves during different life stages (50).

Analysis of total and individual SCFAs revealed significant differences between the dietary Zn groups. There are conflicting data on low-Zn-related changes affect the SCFAs concentration in different animal models. Reed et al. observed a significant decrease in the concentration of SCFAs in the Zn deficiency group in chicks (30), while Pieper et al. found the opposite results in pigs (51). In the present study, during the short-term intervention, higher bacterial metabolites were found in the low-Zn-diet animals, especially acetic acid. In terms of genus levels, the short-term inadequate-zinc diet has remarkable positive correlations with various groups of acetate-, propionate-, and butyrate-producing bacteria (*Flavonifractor*, *Alistipes*, *Odoribacter*, *Anaerotruncus*, *Alloprevotella*, *Bacteroides*, and *Parabacteroides*). Previous work showed that cecal SCFAs concentrations, particularly acetate and butyrate, were correlated with cecal pH (52). Therefore, alterations in the cecal environment, such as a reduction in pH through an increased SCFAs level, can result in a notable increase in the Zn bioavailability and uptake (53, 54). Our data suggested that changes in the gut microbiota composition of the Zn-deficient group can increase Zn availability to combat the Zn-limited conditions. However, it is known that high-dose acetate can cross the blood-brain barrier and reach the hypothalamus, decreasing appetite and nutrition intake (55). For growth parameters, feed intake was suppressed in the Zn-starvation mice, which indicated that the increase in acetic acid production is one of the reasons for feed intake reduction. Also, the negative effects of excess Zn level on SCFAs concentration are in line with the previous findings on piglets (56, 57). Surprisingly, during the long-term intervention, high-Zn-fed mice showed the lowest concentration of SCFAs, except valeric acid and hexanoic acid. Combined with the genus, although the long-term 150-mg/kg Zn diet showed the highest abundance of *Bifidobacterium*, which indirectly contributes to the production of SCFAs (58), it inhibited the enrichment of SCFA-producing bacteria more than the overdosed diet (600 mg/kg). Consistent with the result for SCFAs, the KEGG pathway “lipid metabolism” was also significantly decreased in the high-Zn-fed mice. In addition, cecal SCFAs production decreases along the colon as a function of distance from the cecum due to absorption by colonocytes for energy or for use in cholesterol, fat, and sugar metabolism (59). Moreover, SCFAs were found to play essential roles in inflammation suppression, thus promoting the integrity of the intestinal epithelium (60). The fractional colon inflammatory cell infiltration was partially shown in the 8-week high-Zn-fed mice. We speculated that long-term exposure to a high Zn level increases susceptibility to colitis by reducing short-chain fatty acids and decreasing microbial diversity. Depressed levels of SCFAs and decreased α-diversity have been described in the stool of IBD patients (61, 62). In addition, the long-term excess-Zn diet showed a dramatic decrease in valerate production. As previously described, valerate significantly decreased C. difficile growth in an interventional mouse model of CDI (63, 64). Our data could further support the research by Zackular et al. showing that excess dietary zinc decreases resistance to CDI (18, 65).

With respect to human disease pathways, the individual KEGG terms “drug resistance” and “infection disease” had the highest abundance in the high-Zn-diet group and the lowest abundance in the low-Zn-diet group. It was observed that 150 mg/kg Zn contributes to an enrichment of the potential pathobiont with drug resistance genes. In agreement with the literature, subinhibitory levels (below the MICs) of heavy metals (Cu, Ag, Cr, and Zn) increased antibiotic-resistant bacteria and antibiotic resistance genes (ARGs), which in turn contribute to the resistance phenomena via facilitating the horizontal transfer of ARGs (66). To further discusses the effect of intestinal zinc on human diseases, we assessed differences in probiotics and, potentially, pathobionts among the top 20 species. Lactobacillus reuteri is a well-studied probiotic bacterium (67); the decrease in the abundance of L. reuteri in humans in the past decades is correlated with an increase in the incidences of inflammatory diseases over the same period (68). In our study, L. reuteri was substantially influenced by imbalanced Zn diets, where the excessive Zn diet groups had the lowest proportion of L. reuteri in the 4- and 8-week interventions. In contrast, it has also been reported that L. reuteri was less affected by pharmacological doses of ZnO (2,425 mg/kg) in pigs (56). This could be due to the ZnO therapy for weaned piglets decades ago, which promoted zinc resistance in intestinal bacteria (69). The above-described findings would support a forward selection of L. reuteri in pigs with the specific evolutionary adaptation through dietary zinc amounts. Helicobacter hepaticus is the enteric or enterohepatic *Helicobacter* species (70); outgrowth of H. hepaticus is related to chronic hepatitis, IBD, colitis, and gallstones (71). A high proportion of H. hepaticus was observed in the short-term low-Zn diet and long-term high-Zn diet groups. Very little is known about the relationship between the dietary Zn levels and the abundance of H. hepaticus. A previous study showed that Helicobacter pylori infection has no significant effect on serum zinc levels among children (the gastric *Helicobacter* species) (72). However, Tran et al. found that short-term zinc supplementation (Zn acetate, 1 mg/ml; ZnSO_4_, 5 mg/ml) attenuated Helicobacter felis-induced gastritis in mice (the gastric *Helicobacter* species) (73). Therefore, the correlation between *Helicobacter* species and the Zn level needs further research.

Previous studies tended to utilize serum Zn, nail Zn, and Zn-related genes/proteins (enzymes, inflammatory cytokines, transporters, and binding proteins) to reflect dietary Zn intake (74–76), whereas in our research, the relative abundance of *Melainabacteria* (phylum) and *Desulfovibrio* sp. ABHU2SB (species) appears to be efficacious in differentiating the deficient, normal, or high/excess Zn status in mice. Regarding the Zn-related gene/protein expression, these biomarkers may not be sensitive enough to reveal differences in zinc status in a relatively short-term Zn intervention and could not distinguish between Zn deficiency and Zn excess (77). Although serum Zn is currently the most reliable predictor, the inherent problems of its measurement and interpretation may disrupt the sensitivity and specificity of serum zinc (20). The development of sequencing techniques and modeling algorithms facilitates the predictions of the risk of malnutrition and disease outbreaks by microbiota, which is one of the goals of microbiome research (78). Specifically, intestinal microbial organisms are directly exposed to dietary Zn and easily affected by altered Zn intake (41, 79). Spenser and coworkers found that Clostridium indolis and unclassified *Bacteroidales* were inversely correlated with the final body weight and the dietary Zn adequacy in chickens, which provided evidence for the potential effectors of the chronic Zn-deficient phenotype (30). However, the relative abundance of *Clostridium* species (*Clostridium* sp. ASF502, Clostridium leptum, etc.) was less dependent on the dietary Zn over the 4-week and 8-week intervals in mice. Our results showed that *Melainabacteria* (phyla) and *Desulfovibrio* sp. ABHU2SB (species) were markedly correlated with serum Zn levels in both the short-term and long-term interventions and responded to Zn imbalance in a dose-dependent fashion. *Melainabacteria*, a sibling phylum identified in 2013, is identified in groundwater, wastewater treatment plants, and herbivorous mammal and human guts and is also known to synthesize vitamins B and K, which suggests they are beneficial bacteria to their hosts (80). ROC analysis showed that the AUCs of *Melainabacteria* for distinguishing low Zn levels and high/excess Zn levels were both higher than 0.85. A recent study reported that *Melainabacteria* was ranked the second in abundance in fresh tap water (no Zn detected) yet declined in rank in poststagnation water samples (Zn detected), which is in line with our study of mice (81). With regard to *Desulfovibrio* sp. ABHU2SB, the predicted accuracy was higher than 0.85 for Zn deficiency status and higher than 0.8 for Zn excess status. However, the function of *Desulfovibrio* sp. ABHU2SB and the influence of Zn on *Desulfovibrio* sp. ABHU2SB have not been well studied. Recent studies reported that the species *Desulfovibrio* sp. ABHU2SB thrives in experimental autoimmune uveitis rat and type 2 diabetes animal model KKAy mice (82, 83). Future studies are needed to investigate the mechanism of dietary Zn level on the modulation of these potential microbial markers in order to elucidate new roles for this specific microbe in the etiology and/or progression of Zn imbalance.

Collectively, these data revealed that dietary Zn is an essential mediator of microbial community structure and that both Zn deficiency and Zn overdose could generate a dysbiosis in gut microbiota. Moreover, specific microbial biomarkers of Zn status were identified and correlated with serum Zn level. Our study found that a short-term low-Zn diet (0 mg/kg) and a long-term high-zinc diet (150 mg/kg) had obvious negative effects in a mouse model. Thus, these results indicated that the provision and duration of supplemental Zn should be approached with caution.

## MATERIALS AND METHODS

### Animals.

All experimental protocols of this study were approved by the Animal Ethics Committee of Zhejiang University in accordance with the relevant regulations and laws. Studies were conducted on 3-week-old male C57BL/6 mice that were maintained on a 12-h light/dark cycle under a temperature-maintained room (20 ± 3°C) and caged in stainless steel cages with free access to deionized water.

### Dietary modulation.

Diets containing altered Zn levels were synthesized by SLAC Experimental Animals LLC (Data set S1). Zn was added as carbonate salts, using the AIN-93M standardized diet (84). We aimed to model the relative amount of imbalance Zn supplement the children intestine might be exposed to based on the amount of Zn that human infants are exposed to. The low-Zn diet had 0 mg/kg Zn added back to the base diet, which was intended to model Zn starvation in infants. The control-Zn diet, supplemented with 30 mg/kg Zn, met the recommended value of dietary zinc for mice. The high-Zn diet contained 150 mg/kg Zn, which is 5-fold the amount of Zn in standard mouse chow. It was designed to model the Zinc consumption of many infants, which normally ranges from 4 to 12 times their adequate intake (7–9). The excess-Zn diet, with 600 mg/kg Zn added, was designed to model Zn-overload intake. A total of 64 mice were fed altered Zn diets starting at 3 weeks of age, and diets were maintained for 4 weeks (short-term zinc intervention) or 8 weeks (long-term zinc intervention) to model children from weaning to puberty and maturity (*n* = 8).

### Sampling.

During the treatment period, the body weights, feed intake, and water intake of the animals were monitored regularly. At the end of the study, mice were fasted for 8 h before being euthanized with chloral hydrate followed by cervical dislocation, and blood was collected from sedated mice by retro-orbital plexus before sacrifice. Fresh cecal chymus samples from mice were snap-frozen in liquid nitrogen and stored at −80°C until analysis.

### Histological analysis of the colon.

Briefly, the colon was collected and fixed in 10% phosphate-buffered saline (PBS)-buffered formalin and embedded in paraffin. The 0.2-mm paraffin sections were stained with hematoxylin and eosin (H&E). Photomicrographs were obtained using an inverted microscope (Olympus CKX41, Japan).

### Zn quantification.

Serum Zn levels was assessed with a zinc assay kit (E011-1-1; Jiancheng Bioengineering Institute, Nanjing, China); serum Zn forms a colored complex with 5-Br-PAPS in the reagent at room temperature, and the depth of the color is proportional to the zinc ion concentration. The serum Zn levels were positively correlated with dietary Zn in both the 4- and 8-week intervention groups (Fig. S3).

### DNA extraction and 16S rRNA gene sequencing.

Total genome DNA from samples was extracted using the cetyltrimethylammonium bromide (CTAB)/SDS method. Illumina MiSeq sequencing and general data analysis were performed by a commercial company (Novogene, Beijing, China). The 16S rRNA gene sequence data have been deposited in the NCBI SRA under no. PRJNA749497.

### Bacterial metabolites.

Microbial metabolite analysis was performed for randomly selected mice (*n* = 6/group) representing both sets of cages in each diet group. Samples were treated as follows: 30 mg of cecal chymus was put in a 2-ml glass centrifuge tube, 900 μl 0.5% phosphoric acid was added, and the tube was shaken for 2 min and then centrifuged at 14 000 × *g* for 10 min. Then, 800 μl of the supernatant was extracted, and the same amount of ethyl acetate was added to the extract. Next, 600 μl supernatant of the extract was mixed with 500 μM (final concentration) of internal standard (4-methylpentanoic acid) before injection. The samples were separated on an Agilent DB-WAX capillary column (30 m × 0.25 mm ID × 0.25 μm) gas chromatography system. The temperature programming was as follows: the initial temperature was 90°C and remained as such for 3 min. The temperature increased at 10°C/min up to 120°C and then increased at 25°C/min up to 250°C and remained there for 20 min. The carrier gas was helium, and the carrier gas velocity was 1.0 ml/min. A quality control (QC) sample was used for testing and evaluating the stability and repeatability of the system. An Agilent 7890A/5975C gas chromatography-mass spectrometer was used for analysis. The temperatures of the injection port and transmission line were 250°C and 230°C, respectively. The electron bombardment ionization (EI) source, selected ion monitoring (SIM) scanning mode, and electron energy were 70 eV. The MSD ChemStation software was used to extract chromatographic peak area and retention time. The content of medium- and long-chain fatty acids in the sample was calculated by plotting the curve. The quality control samples were processed together with the biological samples. The detected metabolites in pooled samples with a coefficient of variation (CV) less than 30% were denoted reproducible measurements.

### Analysis of the microbiota.

The reads of the paired ends were assigned to the samples based on their unique barcodes and truncated by cutting off the barcode and primer sequence. Paired-end reads were merged with FLASH v1.2.7 (85). According to the quality-controlled process of QIIME v1.9.1 (86), the raw labels were quality filtered under specific filtering conditions in order to obtain high-quality clean tags (87). By detecting chimeric sequences using the UCHIME algorithm (88), the tags were compared with a reference database (Silva database), and finally, valid tags were obtained and then the chimeric sequences were removed (89). Sequences analysis was performed with UPARSE v7.0.1001 (90). Sequences with similarity of ≥97% were assigned to the same operational taxonomic units (OTUs). The representative sequence of each OTU was screened for further annotation. Subsequently, the alpha diversity and beta diversity were analyzed based on this standardized output data.

For biomarker identification, linear discriminant analysis effect size (LEfSe) analysis (http://huttenhower.sph.harvard.edu/galaxy/) was used to determine differences in categorical component classification between the 4 cohort groups. Candidate biomarkers with linear discriminant analysis (>3) were selected as the effective biomarkers. The discriminatory power of the biomarkers was assessed by plotting receiver operating characteristic (ROC) curves and calculating the area under the ROC curve using Prism 8.

To further understand the specific functions of each group of bacteria, 16S high-throughput sequencing data were used to classify the OTUs through the SILVAngs platform based on the SILVA database. The 16S copy number was then standardized according to the National Center of Biotechnology Information genome annotation. Finally, the prediction of the microbial community function was realized by constructing a linear relationship between the SILVA classification and pronuclear classification in the KEGG database.

### Statistical analysis.

The statistical analysis was carried out using SPSS v22.0 (IBM, Inc.). All values were expressed as the mean ± standard deviation (SD). Comparisons between multiple groups were analyzed by one-way analysis of variance (ANOVA) with a post hoc Bonferroni test. *P* values of <0.05 were considered to be a significant difference. For correlation analysis, Spearman and Pearson analyses were performed.
