# Supplementary material for: Effect of Long-Term and Short-Term Imbalanced Zn Manipulation on Gut Microbiota and Screening for Microbial Markers Sensitive to Zinc Status
Source: Microbiol Spectr. 2021 Nov 3;9(3):e00483-21. doi: 10.1128/Spectrum.00483-21 (PMC8567254; doi:10.1128/Spectrum.00483-21)
Supplement: SUPPLEMENTAL FILE 1 — Supplemental material. Download SPECTRUM00483-21_Supp_1_seq11.pdf, PDF file, 2.5 MB [file spectrum00483-21_supp_1_seq11.pdf]

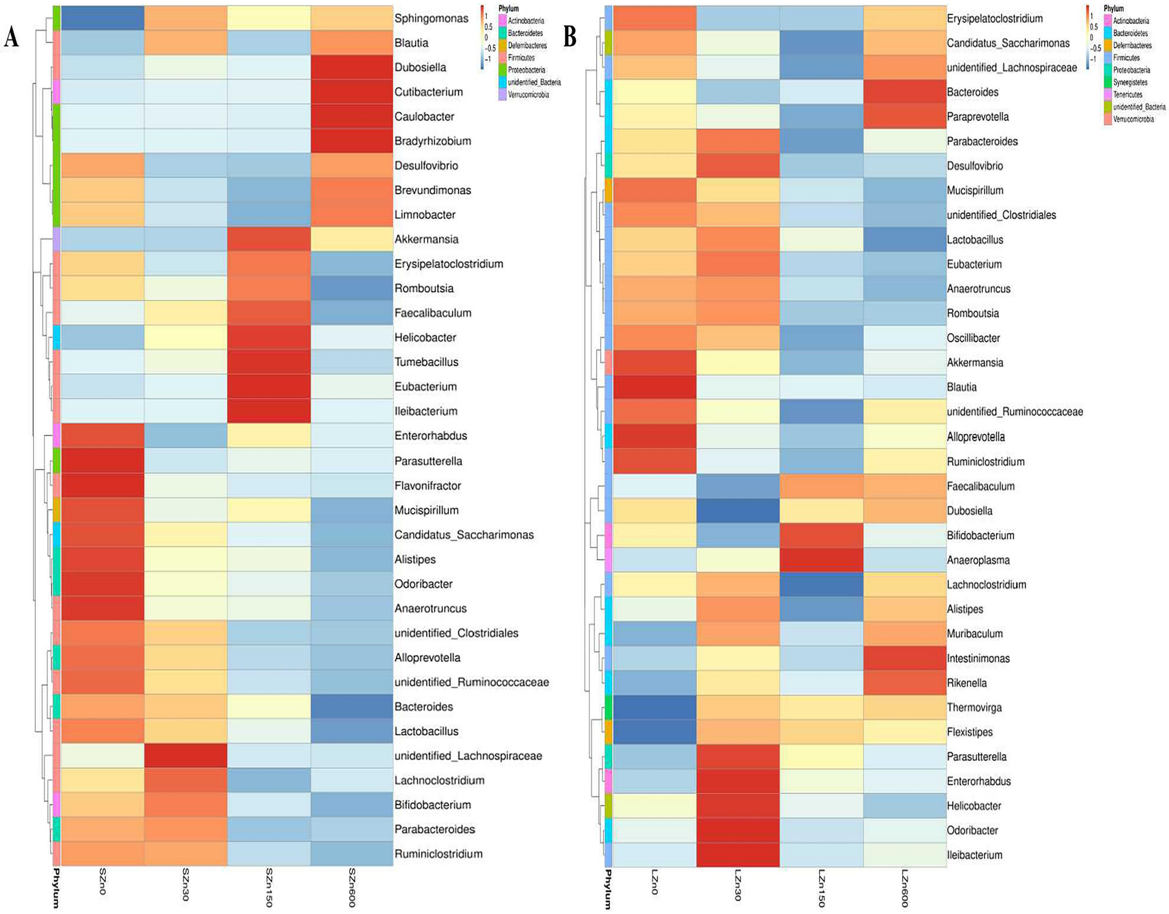

Fig.S1 Imbalance dietary zinc disturbs the microbial community structure in the mice cecum at genus level. (A) The top 30 microbial population at genus levels in the cecum of mice from the short-term 0 mg/kg Zn intervention (SZn0), short-term 30 mg/kg Zn intervention (SZn30), short-term 150 mg/kg Zn intervention (SZn150) and short-term 600 mg/kg Zn intervention (SZn600). (B) Relative abundance (top 10) of bacterial genus in the cecum of mice from the long-term 0 mg/kg Zn intervention (LZn0), long-term 30 mg/kg Zn intervention (LZn30), long-term 150 mg/kg Zn intervention (LZn150) and long-term 600 mg/kg Zn intervention (LZn600).

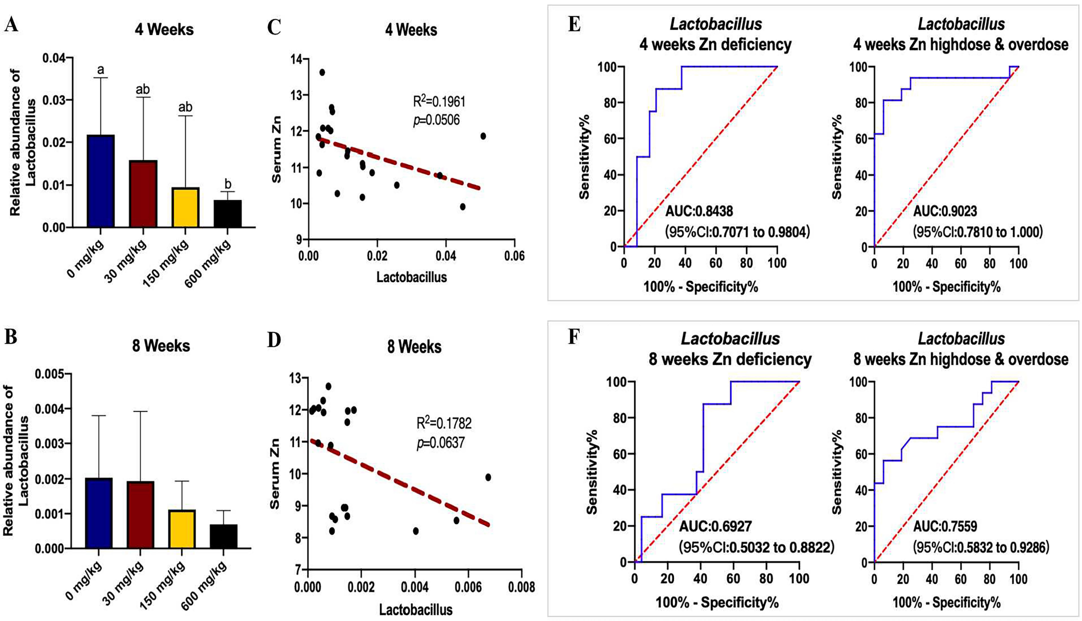

Fig.S2 Prediction of Imbalanced Zn Based on Bacterial Markers in Gut. The relative abundance of *Lactobacillus* in altered Zn groups in (A) 4-week and (B) 8-wewk intervention. correlation analysis between the proportion of *Lactobacillus* and serum Zn levels in (C) 4weeks and (D) 8 weeks. Prediction (Zn defeciency/Zn highdose & overdose) of *Lactobacillus* in the microbiome of altered Zn-fed mice in (E) 4weeks and (F) 8 weeks.

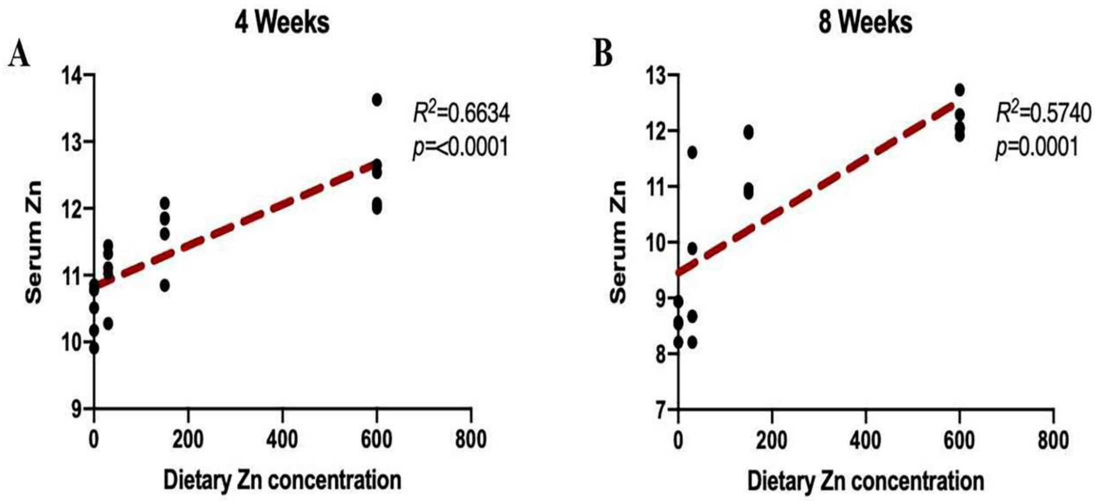

Fig.S3 Correlation analysis between dietary Zn and serum Zn. The serum Zn levels were positively correlated to dietary Zn both in (A) 4-week and (B) 8-wewk intervention.

Group	A	observed-delta	expected-delta	Significance
SZN0-SZN150	0.1117	0.3083	0.3471	0.002
SZN0-SZN30	0.1206	0.2602	0.2959	0.002
SZN30-SZN150	0.09095	0.2971	0.3268	0.001
SZN0-SZN600	0.216	0.2537	0.3236	0.001
SZN150-SZN600	0.1084	0.2906	0.3259	0.001
SZN30-SZN600	0.1983	0.2425	0.3025	0.001
LZN30-LZN600	0.2569	0.3017	0.4059	0.001
LZN30-LZN150	0.3186	0.288	0.4227	0.001
LZN150-LZN600	0.12	0.3173	0.3605	0.003
LZN0-LZN30	0.2424	0.2967	0.3916	0.001
LZN0-LZN600	0.107	0.3259	0.3649	0.001
LZN0-LZN150	0.2066	0.3123	0.3936	0.001

Table.S1 Microbiological inter-group difference analysis using a non-parametric analyses of multi-response permutation procedure (MRPP). SZn0, SZn30, SZn150 and SZn600 represent short-term 0 mg/kg Zn intervention, short-term 30 mg/kg Zn intervention, short-term 150 mg/kg Zn intervention and short-term 600 mg/kg Zn intervention. LZn0, LZn30, LZn150 and LZn600 represent long-term 0 mg/kg Zn intervention, long-term 30 mg/kg Zn intervention, long-term 150 mg/kg Zn intervention and long-term 600 mg/kg Zn intervention.

	0 mg/kg Zn		30 mg/kg Zn		150 mg/kg Zn		600 mg/kg Zn	
%	gm	kcal	gm	kcal	gm	kcal	gm	kcal
Protein	20.0	20.5	20.0	20.5	20.0	20.5	20.0	20.5
Carbohydrate	66.3	68.0	66.3	68.0	66.2	68.0	66.2	68.0
Fat	5.0	11.5	5.0	11.5	5.0	11.5	5.0	11.5
Total		100.0		100.0		100.0		100.0
kcal/gm	3.90		3.90		3.90		3.90	
Ingredient	gm	kcal	gm	kcal	gm	kcal	gm	kcal
Egg Whites,Spray Dried	200	800	200	800	200	800	200	800
Casein	0	0	0	0	0	0	0	0
DL-Methionine	0	0	0	0	0	0	0	0
Corn Starch	150	600	150	600	150	600	150	600
Sucrose	502.6	2010.4	502.6	2010.4	502.6	2010.4	502.6	2010.4
Cellulose,BW200	50	0	50	0	50	0	50	0
Corn Oil	50	450	50	450	50	450	50	450
Mineral Mix S190401(No Added Zn)	35	0	35	0	35	0	35	0
Mineral Mix S190401A(No Zn,Fe)								
Mineral Mix S10001		0		0		0		0
Vitamin Mix V10001	10	40	10	40	10	40	10	40
Choline Bitartrate	2	0	2	0	2	0	2	0
Biotin,1%	0.4	0	0.4	0	0.4	0	0.4	0
zinc Carbonate,Basic	0	0	0.053	0	0.248	0	1.099	0
54.6%Zinc								
Total	1000	3900	1000.1	3900	1000.298	3900	1001.149	3900

Supplementary Data Set.S1 Composition and nutrient levels of the experimental diets.
